# THE EFFECT OF RESILIENCE ON USING COMMUNITY ORGANIZATIONS AMONG KOREAN AMERICAN OLDER ADULTS DURING COVID-19

**DOI:** 10.1093/geroni/igad104.2341

**Published:** 2023-12-21

**Authors:** Eunhye Kim, Hyesu Yeo, Y Joon Choi

**Affiliations:** Augusta University, Evans, Georgia, United States; University of Georgia, Athens, Georgia, United States; Georgia State University, Atlanta, Georgia, United States

## Abstract

**Objectives:**

This study aimed to examine how resilience and perceived racial discrimination are associated with receiving support from ethnic community organizations (ECO) among Korean American older adults during COVID-19.

**Background:**

With the vulnerability of Korean American older adults and increasing racial discrimination, ethnic community organizations’ roles are important during COVID-19. Although previous studies have proven the association of resilience and racial discrimination with older adults’ well-being, there is little research on how those factors impact using ECO.

**Methods:**

A cross-sectional survey was conducted with 132 first-generation Korean American adults aged 50 or older. A logistic regression analysis was conducted to determine which factors, including resilience, discrimination, age, gender, marriage, health, residence years, Language, and income, within the model made them more likely to receive support from ECO.

**Results:**

Results showed that a high level of resilience was negatively associated with the use of ECO[OR 0.93, CI 0.87, 0.99]. Korean American older adults who were female [OR 5.60, CI 1.44, 21.50] and lower income [OR 4.42, CI 1.35, 14.48] were more likely to receive support. Those who were healthy [OR 2.15, CI 1.08, 4.26] and had lived longer in the U.S.[OR 1.07, CI 1.00, 1.14] were more likely to use ECO.

**Conclusion:**

Findings from the study highlight the importance of improving and expanding support for vulnerable ethnic minority older adults in order for them to access services and develop effective coping strategies during the health crisis.

